# Influence of cosmetic formulations on the skin's circadian clock

**DOI:** 10.1111/ics.12623

**Published:** 2020-06-05

**Authors:** S. Hettwer, E. Besic Gyenge, B. Obermayer

**Affiliations:** ^1^ RAHN AG Dörflistrasse 120 Zürich 8050 Switzerland

**Keywords:** circadian rhythm, epidermal jetlag, cosmetic active

## Abstract

**Objective:**

The circadian rhythm was set into focus by awarding the Nobel Price of Physiology/Medicine to Jeffrey Hall, Michael Rosbash and Michael Young in late 2017. Numerous publications elucidated the molecular mechanisms driving the circadian biorhythms of our body, peripheral organs and each single cell. However, there is minor knowledge on the circadian rhythm of the skin, which has its own peripheral circadian clock in contact with cosmetic formulations. The skin's epidermal clock is excessively influenced by environmental factors like UV radiation or modern lifestyle, which may induce epidermal jetlag. Here, we give an overview on the current knowledge about the epidermal circadian clock and provide a cosmetic solution to protect and preserve the biorhythm of the skin.

**Methods:**

Quantitative RT‐PCR to analyse the gene expression of circadian clock genes and the downstream DNA repair gene OGG1 in keratinocytes irradiated with UV‐B. In vivo study to determine skin parameters dependent on the circadian cycle and interference of cosmetic formulations to them by assessment of morning and evening values at each measurement day after 28, 56 and 84 days of the study.

**Results:**

UV‐B irradiation leads to a pronounced delay in circadian clock and downstream gene expression which interferes in the proper function of epidermal stem cells and as thus skin function. The use of a cosmetic active ingredient prevents cyclobutane pyrimidine dimer formation, protects epidermal stem cells and resets the circadian gene expression. It preserves the circadian changes in skin hydration, reduces daily fluctuations of skin redness and strengthens the skin barrier.

**Conclusion:**

The skin has its own circadian biorhythm to gain full functionality. Interruption of this oscillation will lead to functional impairments. Here we show a cosmetic solution to protect and preserve the skin's circadian rhythm. DNA protection, ROS elimination and stimulation of circadian gene expression seem to be crucial to keep the skin in balance.

## Graphical abstract

The circadian Rhythm of all cells, including skin cells, is managed by a sophisticated steering of gene expression of only few important Clock genes.

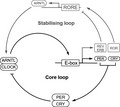

## Résumé

**Objectif:**

Le rythme circadien a été mis en lumière par l'attribution du prix Nobel de physiologie/médecine à Jeffrey Hall, Michael Rosbash et Michael Young fin 2017. De nombreuses publications ont élucidé les mécanismes moléculaires qui régissent les biorythmes circadiens de notre corps, des organes périphériques et de chaque cellule. Cependant, il existe peu de connaissances sur le rythme circadien de la peau, qui possède sa propre horloge circadienne périphérique, avec les formules cosmétiques. L'horloge épidermique de la peau est excessivement influencée par des facteurs environnementaux comme les rayons UV ou le mode de vie moderne, qui peuvent induire un "décalage horaire de l'épiderme". Nous donnons ici un aperçu des connaissances actuelles sur l'horloge circadienne épidermique et proposons une solution cosmétique pour protéger et préserver le biorythme de la peau.

**Méthodes:**

Test RT‐PCR quantitatif pour analyser l'expression des gènes de l'horloge circadienne et du gène de réparation de l'ADN OGG1 en aval dans des kératinocytes irradiés aux UV‐B. Étude in vivo pour determiner les paramètres cutanés dépendant du cycle circadien et l'interférence des formules cosmétiques sur ces paramètres par évaluation des résultats du matin et du soir à chaque jour de mesure après à 28, 56 et 84 jours d'étude.

**Résultats:**

L'irradiation aux UV‐B entraîne un retard prononcé de l'horloge circadienne et de l'expression des gènes en aval, ce qui interfère avec le bon fonctionnement des cellules souches épidermiques et donc en conséquence avec la fonction de la peau. L'utilisation d'un principe actif cosmétique empêche la formation de dimère de cyclobutane pyrimidine, protège les cellules souches épidermiques et rétablit l'expression génique circadienne. Il préserve les changements circadiens dans l'hydratation de la peau, réduit les fluctuations quotidiennes des rougeurs cutanées et renforce la barrière cutanée.

**Conclusion:**

La peau a son propre biorythme circadien pour acquérir une assurer une parfaite fonctionnalité. L'interruption de cette oscillation entraînera des troubles fonctionnels. Nous présentons ici une solution cosmétique pour protéger et préserver le rythme circadien de la peau. La protection de l'ADN, l'élimination des ROS et la stimulation de l'expression des gènes circadiens semblent être cruciales pour maintenir l'équilibre de la peau.

## Introduction

### The central circadian clock

All of the body's biofunctions are adapted to the 24 h cycle of night and day. For proper functioning of this biorhythm, the circadian timing system, all the circadian clocks in the body needs to be synchronized with one another. To achieve this, a small number of neurons in the suprachiasmatic nucleus (SCN) in the brain function as the central pacemaker of the entire body. The cells of the SCN receive direct input from the retina allowing the determination of light and dark phases of day and night, respectively [1]. To regulate distant body parts, diverse pathways are used, among them autonomic neural connections [2] and hormones [3, 4, 5]. The system requires the presence of peripheral circadian clocks in all cells of the body, which are synchronized with the master clock's output signals [6]. If this is missing, the rhythm in most tissues gradually damp out within few days [7]. The most obvious situation where we encounter the power of the circadian rhythm is the jetlag because of long‐distance travelling. Interestingly, the central pacemaker in the SCN very rapidly recalibrates to the new day/night situation although adjacent areas in the brain or peripheral body tissues need at least 8 days to be fully in phase with the master clock again [8].

### The biochemical steering of the circadian clock

**Figure 1:**
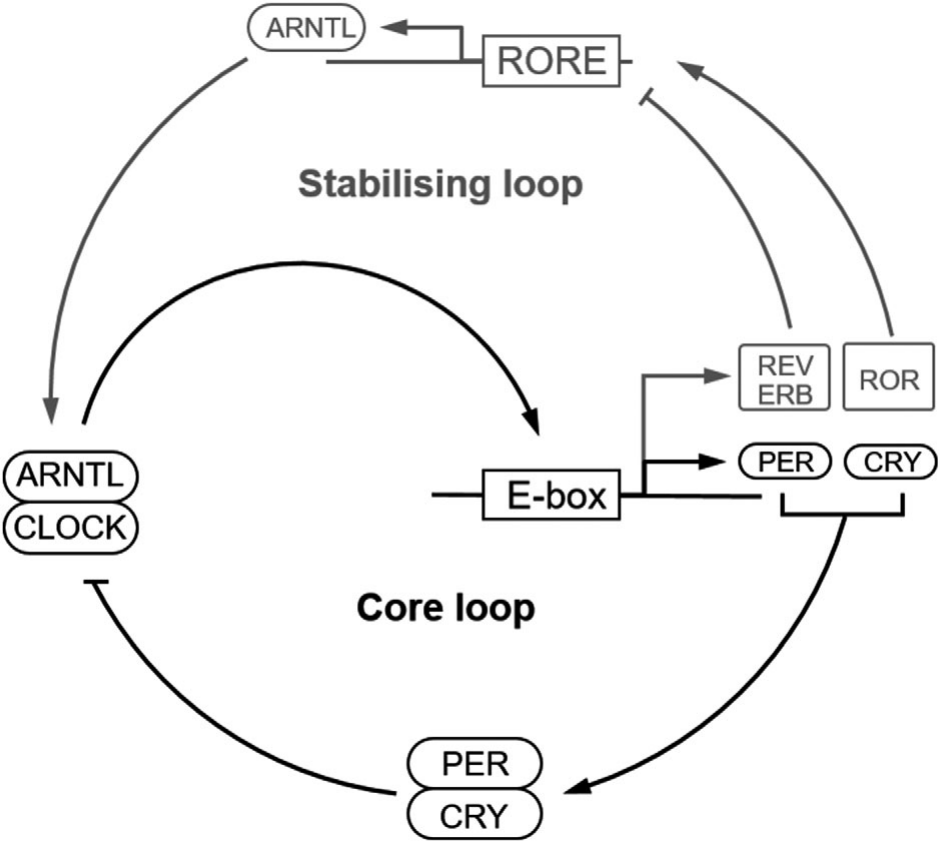
The key players of the intracellular circadian clock are controlled in a feedback core loop and a stabilizing loop [6]. Circadian controlled genes are switched on by the ARNTL/CLOCK complex, among them the negative regulators PER and CRY with direct inhibitory effect on the ARNTL/CLOCK complex and REV‐ERB blocking expression of ARNTL. This stabilizing loop is balanced by the expression of the positive regulator ROR. For more information, see text.

On molecular level, the circadian clock in the SCN neurons as well as in peripheral cells is set by the controlled expression and degradation of only a few key proteins (Fig. 1). In the SCN, light induces the expression of PER and CRY by depolarization of the neuronal membrane, via phosphorylation of CREB, binding to a CREB response element (CRE) upfront of the promoter of PER and CRY. To enable oscillated expression, the proteins ARNTL and CLOCK facilitate gene expression by binding to an E‐box element [1].

In a core loop, the proteins CLOCK and ARNTL (formerly called BMAL1) form a heterodimer and act as transcription factors enhancing the expression of genes containing an E‐box enhancer element [6]. Among those are genes important for light‐phase activities but also for the negative feedback proteins PER and CRY. They are translated and accumulate in the cytoplasm first, as staining of the proteins shows [7]. When forming a PER/CRY heterodimer, they translocate into the nucleus and repress the enhancer complex CLOCK/ARNTL. As a consequence, they repress their own transcription. By controlling the timed degradation of the PER/CRY complex by the proteasome, the 24‐h cycle is ensured [9]. To control the periodic appearance of the CLOCK/ARNTL enhancer complex, a stabilizing loop expressing the suppressive REV‐ERBα/β and the enhancing RORα/β is switched which regulate the concerted expression of ARNTL by competing to binding to the transcription enhancer site RORE on the promoter of ARNTL [6]. It appears that periodic expression for ARNTL is much stronger than for CLOCK. The synchronization signal of the SCN ensures the proper calibration of the cellular circadian clocks in the entire body.

### The circadian clock in the epidermis

As every organ, also the skin as a peripheral tissue has its own circadian rhythm [10, 11, 12]. As the epidermis has a constant cell turnover, the correct timing of events is crucial. The basal layer provides the epidermis with a timed supply of new cells. The epidermal basal layer stem cells or progenitor cells are supposed to divide once a day around the evening or early night pushing the cells on top to the outside of the epidermis. This process will induce differentiation of the keratinocytes in respect to the current location in the stratum spinosum or stratum granulosum and stratum corneum, respectively. The calcium gradient plays an important role in that a low calcium level will set cultivated normal human epidermal keratinocytes into the stage of basal layer progenitors although high calcium will provoke differentiation into granulocytes [13]. The circadian clock of keratinocytes depends on their positioning in the skin. Genes for keratinocyte differentiation are expressed during late night and early morning which means that the skin barrier repair starts in the morning hours, in line with higher TEWL values which can be measured in the morning compared to the evening [14]. Genes for cell proliferation and cell division in the basal layer are expressed late afternoon and in the evening. On macroscopic level, this translates to the strongest skin barrier and the most hydrated skin in the afternoon whereas the least hydrated and least strong skin barrier appears in the deep night/early morning [12]. Such kinds of skin conditions typically lead to itchy skin but this is not recognized during the sleep.

### UV radiation impacts the circadian clock

Human basal epidermal stem cells prepare for cell division in the S‐phase of the cell cycle by DNA synthesis during daytime [6]. As a diurnal being, active during the day, this seems to be contradictory as the sun's UV radiation will provoke numerous DNA damages either directly in forming cyclobutane pyrimidine dimers or indirectly by generation of DNA attacking ROS [15]. As a consequence, the human DNA repair machinery evolved to be highly efficient in repairing light‐induced DNA damages during the day and as such, during the S‐phase of the cells [16]. Therefore, the expression of the corresponding nucleotide excision repair enzymes peak their expression during daytime, steered by the circadian rhythm. In concordance, the susceptibility to develop a sunburn or even melanoma is higher when exposed to sun in the evening than in the morning [16, 17]. In principle, the skin has learned to protect itself against UV radiation by using melanin. The system works very well when the skin is adapted but nowadays, this protective measure seems to be insufficient. To be fully protected, the skin has to be treated with cosmetic formulations containing a proper sun protection factor (SPF). There is a good reason to protect the skin against UV radiation as not only DNA damage and the subsequent development of an erythema, induced by inflammatory response, are the results. In addition, it was shown that UV‐B radiation will disrupt circadian and downstream gene expression by up to 24 h [18]. As such, whereas fighting against DNA damage and inducing inflammation, the keratinocytes are blocked in their cell cycle and the epidermis cannot use its routine program to regenerate according to the circadian rhythm.

### Epidermal Jetlag

UV‐B radiation is the major daily impact our skin faces in terms of dysregulation of the circadian clock gene expression [19]. Although this can be handled by applying a proper SPF, other damaging radiations like blue light may still be able to induce ROS and indirect DNA damage, which has to be repaired. However, for the circadian clock of the skin, another aspect of blue light is important. Blue light is the quality of light, which sets the clock in the SCN. The modern lifestyle prevents the proper setting of the central circadian clock as we may get up before sunrise and we go to bed much later than sunset, using unconsciously an amount of artificial (blue) light, which is by far enough to bring the circadian clock out of phase [20]. This can be either just artificial light sources but most notably the use of electronic devices emitting predominantly blue light. As the central clock sets the peripheral clocks, the skin is one organ to suffer from this situation. The finely balanced clockwork of cell division and regeneration gets out of pace which results in uncomfortable skin conditions at times we usually do not recognize them. We may call this Epidermal Jetlag which obviously also occurs when faced to a time shift because of long haul flights.

### A cosmetic active to preserve the skin's circadian clock

What are the possibilities to protect the skin cells from Epidermal Jetlag? Living a life with the sun but avoiding direct sunlight to the skin would be the obvious solution. However, this cannot be integrated in our all days life, easily. Especially in summer time, the UV‐B radiation from the sun is the biggest threat. The first defence line would be to prevent UV‐B radiation to enter the skin and obviously, a good SPF will do so. This prevents excessive photo‐damage of DNA and replicative stress in the S‐phase. Unfortunately, this will not prevent the formation of DNA‐damaging ROS induced by other sources. Firstly, high energy visible light can excite skin molecules like lipofuscin, AGEs or even vitamins and flavins to create DNA‐damaging ROS [21]. Infrared light can induce ROS by increasing the inflammatory state of the skin [22]. On top, the application of a cosmetic formulation itself can increase the ROS content inside the skin [23]. To counteract, two basic principles can be followed. Firstly, radicals can be scavenged with powerful anti‐oxidants. Secondly, DNA repair can be enhanced by induction of the nucleotide excision repair pathway, in particular the expression of 8‐oxo guanine glycosylase, removing oxidized guanine residues from the DNA which are result of metabolic as well as UV‐related DNA oxidation.

## Material and methods

Primary human keratinocytes from a 37‐year‐old female donor were cultivated in a 12‐h light/dark environment using a white LED lamp. This simulation was not meant to have a major impact on cell physiology but to be able to make reference on the phase the cells are after hormonal synchronization of the circadian cell rhythm. Twenty‐four hours before synchronization, the cells were treated with 0.01 % or 0.001 % active ingredient or with vehicle (DMSO). A synchronized induction of the circadian rhythm was achieved by means of supplementation with 100 nM dexamethasone for 20 min. Afterwards, the cells were irradiated with 15 mJ cm^−2^ UVB. By way of comparison, the UV dose in spring at sea level is in the range 100–180 J cm^−2^, depending on the degree of latitude (i.e. approximately 10 times higher than that of the experiment) [24]. Samples were taken after 6, 18, 30 and 42 h. Gene expression was determined using quantitative reverse transcriptase real‐time PCR (qRT‐PCR). All experiments were done in triplicate with a technical duplicate for each biological replicate. The results were normalized against the endogenous control GAPDH.

The *in vivo* study was performed in accordance with the principles of good laboratory practice (GLP) and good clinical practice (GCP) and in compliance with the quality assurance system requirements. Studies were conducted with respect to World Medical Association in the Declaration of Helsinki. All study participants signed a written informed consent at the beginning of the study.

A double‐blind, placebo‐controlled, randomized study on 44 female subjects with healthy, Caucasian skin aged 35–65 years (average 53.5 years). All test parameters were initially measured before application of any formulation in defined regions of the face at day 0 in the morning (between 8:00 and 10:00) and in the evening (between 20:00 and 22:00) for baseline determination. Subsequent measurements on days 28, 56 and 84 were performed prior to the application of the corresponding formulations twice daily (morning and evening): Placebo INCI: Water (Aqua), Caprylic/Capric Triglyceride, Glycerin, Cetearyl Alcohol, Glyceryl Stearate, Citrate, Sucrose Stearate, Phenoxyethanol, Xanthan Gum, Carbomer, Fragrance (Parfum), Caprylyl Glycol and Sodium Hydroxide. Half of the subjects applied placebo whereas the other half applied the identical formulation containing 3% CELLIGENT^®^.

Skin hydration was measured with a Corneometer^®^ CM 825 at three different positions on the forehead. TEWL was assessed with a Tewameter^®^ TM300 in the central region of the forehead. For determination of skin redness, measurements with a Mexameter^®^ MX 18 in the malar region were performed. The measurements were made under controlled conditions (20°C, 40–60% humidity), and the study participants were adapted to these conditions for an appropriate time.

## Results and discussion

### Gene expression of circadian genes

**Figure 2:**
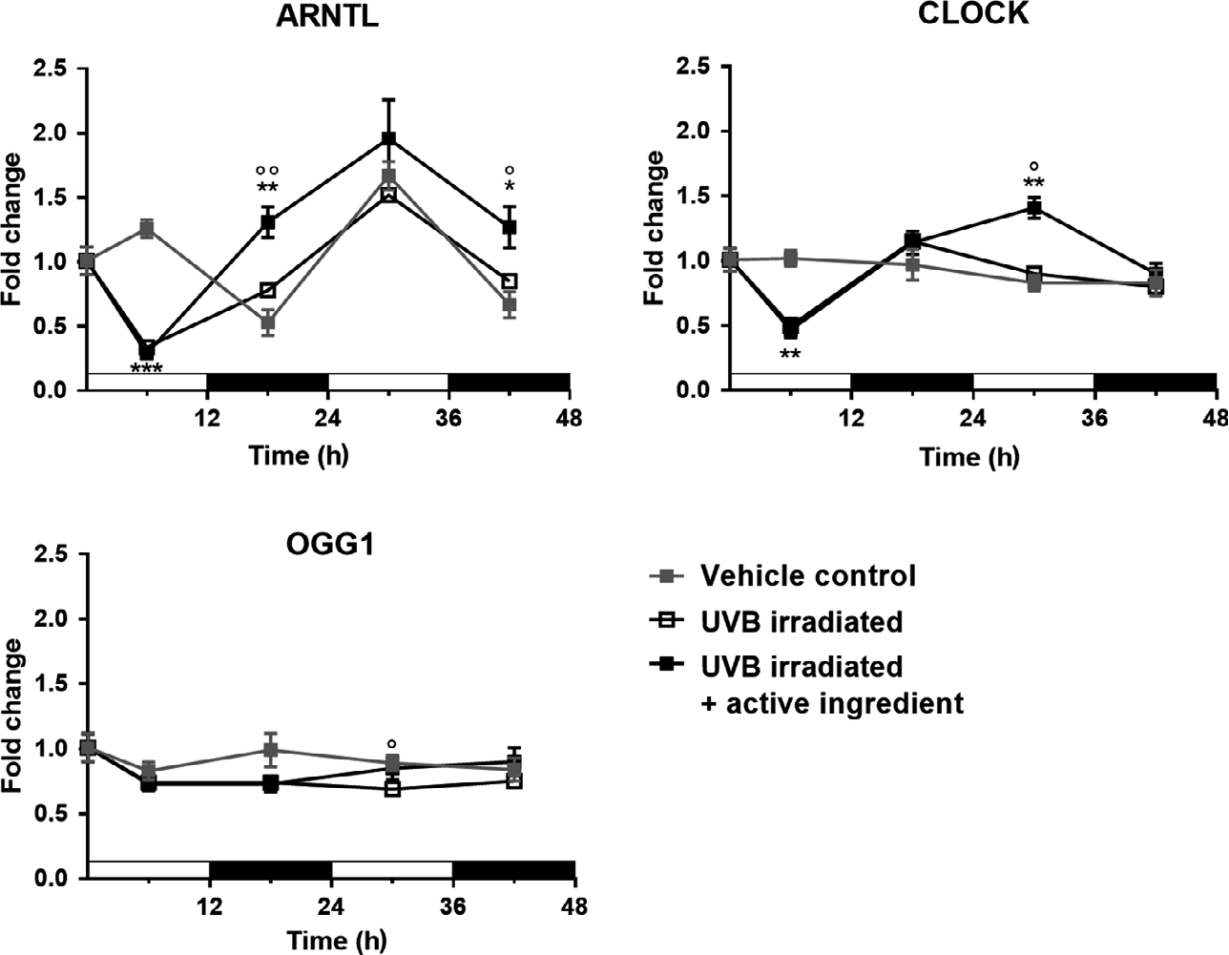
Gene expression of ARNTL, CLOCK and OGG1 in cultivated primary human keratinocytes. After synchronization and UVB irradiation, the expression of ARNTL, CLOCK and to lesser extent the circadian downstream gene OGG1 is downregulated. After 18 h, the expression is back to normal level. Addition of a cosmetic active ingredient accelerates the process for ARNTL and enables periodicity on CLOCK. A trend to higher expression of OGG1 is observed after 30 and 42 h. *: Significance over vehicle control; °: significance over UVB irradiated. One symbol: *P* < 0.05.; two symbols: *P* < 0.01; unpaired Student's *t*‐test. The white bars indicate the day phases whereas the black bars indicate the night phases.

Keratinocytes from skin explants keep their circadian rhythm for several days [25]. In order to achieve a sufficient cell number to facilitate *in vitro* experiments, cells have to be separated from the existing tissue structure, cultivated and expanded. During this time, the circadian oscillation is fading out and has to be restarted again. This can be facilitated by applying a dexamethasone induction that simulates the cortisone peak of the human body in the morning [7]. The result will be the induction of circadian gene expression for a small number of cycles. As can be seen for ARNTL gene expression, the induction resulted in the expected expression peaks in the day phase although expression went down in the night phase (Fig. 2). A full oscillation of two days could be observed. In contrast, an oscillation for the CLOCK gene expression was not observed. This was in agreement with literature [26, 27] and seems to be obvious when considering the mechanism of gene regulation shown in Fig. 1. A similar picture can be seen for PER1 and CRY1. Although PER1 shows a phase‐shifted oscillation compared to ARNTL, CRY1 expression continuously decreased during the experiment. This points to an insufficient activation of the circadian gene expression for some of the corresponding genes, although ARNTL expression showed a good oscillation. The circadian clock downstream gene OGG1 showed a small expression peak after 18 h and did not show obvious changes in expression levels after 30 h. However, the gene expression is in concordance with literature data [26] which shows a peak in OGG1 activity at the end of the night phase. UVB irradiation at 20 min after synchronization of the cells leads to a significant breakdown in gene expression of ARNTL and CLOCK as detected 6 h later. This could not be prevented by addition of the active ingredient. After 30 h, gene expression for ARNTL was completely recovered and dropped again after 42 h in the same way as the untreated control did. Active ingredient treated cells were able to recover gene expression of ARNTL significantly faster after 18 h, and a trend to a higher gene expression after 30 and 42 h was observed. The oscillation was not affected. Also, CLOCK gene expression break down was not prevented by addition of the active ingredient but a significant peaking in gene expression after 30 h was observed, pointing to a consolidated circadian rhythm. PER1 and CRY1 gene expression were largely not affected except a lower gene expression for CRY1 after 18 h where a low level was expected (not shown). For the circadian clock downstream gene OGG1, the expression decreased significantly after UVB irradiation after 30 h, which was not the case when the cells were treated with the active ingredient. A trend to a general higher expression was observed in treated cells after UV‐B irradiation. As reported by Zanello et al. [28], the gene expression directly correlates with the presence of the corresponding proteins.

Although it was shown that the active ingredient can protect cells from DNA damage and enable epidermal stem cells to generate an intact 3D epidermal model after UV‐B irradiation [29], the active was not able to prevent circadian gene knock‐down 6 h after the irradiation impact. This expression knock‐down can be considered as a result of S‐phase‐lock to enable DNA repair and as such eliminate the possibility of generating permanent DNA mutations as a result of the next cell division. To keep the circadian rhythm of (stem‐) cell division, a halt time of 24 h seems to be advantageous until gene expression of ARNTL reaches its maximum again. This halt in the cell cycle seems to be independent on the amount of DNA damage, in that it is still repairable and does not lead to initiation of apoptosis. Treatment with the active ingredient accelerated the recovery from gene expression knock‐down which can be the result of DNA‐damage protection as well as the constant expression of the oxidative DNA‐damage repair gene OGG1. Interestingly, without the external stressor UV‐B irradiation, the active ingredient had no effect on the circadian gene expression of all investigated genes. As such, the ROS eliminating properties of the active ingredient only come into play, when the cells are under considerable stress situations and do not interfere in the normal situation.

Although this *in vitro* gene expression study revealed results in agreement with literature data on changes because of UV‐B irradiation of keratinocytes [18], its major weakness is that only one measurement time point in each light and dark cycle was assessed. As such, it was not possible to determine the exact time points where gene expression was highest or lowest, unlike the graphs implied it. For OGG1 expression, expression peaks could also been missed by the selected time points for investigation. As only keratinocytes from one donor were assessed, the results are rather preliminary and need to be confirmed.

### Circadian changes in human skin parameters

**Figure 3:**
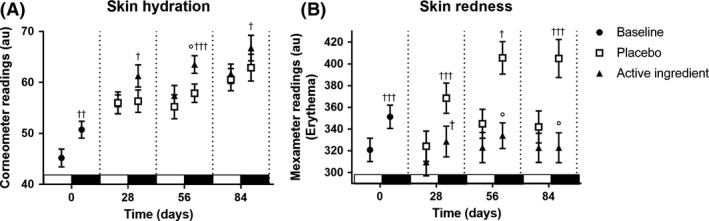
Progression of skin hydration and redness. A: Skin hydration baseline values at day 0 differed significantly by 12%. Improvement of skin hydration using a cosmetic formulation with an active ingredient was significant higher than placebo. B: Skin redness significantly differed by 10% at day 0. Although redness increased significantly for placebo, the active ingredient kept the value constant. †: significance over the corresponding morning value of the same day. °: significance over placebo. One symbol: *P* < 0.05; two symbols: *P* < 0.01; three symbols: *P* < 0.001; unpaired Student's t‐test. The white bars indicate the day phases whereas the black bars indicate the night phases.

In a double‐blind, placebo‐controlled study on 44 female subjects with Caucasian skin, we investigated baseline circadian changes of skin parameters hydration, transepidermal water loss (TEWL) and skin redness before the application of cosmetic formulations. In agreement with literature data, skin hydration was significantly 12 % lower in the morning (between 8 and 10 am) than in the evening (between 8 and 10 pm, Fig. 3A day 0). Investigations with multiple measurements during the day suggest either a 24‐h cycle or even 8‐h cycles in skin hydration [14] leading to a lower skin hydration in the morning than in the evening. For the TEWL, a 24 h circadian rhythm has been observed for the facial region in a way that it is high in the morning and low in the evening, which could be confirmed in our study but without significance (not shown). The circadian rhythm of TEWL seems to be dependent on the body part investigated. Yosipovitch et al. [30] determined a 7‐h shift between the minimal TEWL on the forearm compared to the face. This example shows the difficulties to investigate circadian rhythms on the skin and how to interpret literature data. Skin redness was low in the morning and significantly increased in the evening by 10 % (Fig. 3B, day 0). The reason is a higher microcirculation, either induced by circadian increase of blood pressure or light‐stress induced inflammatory processes [31]. Besides the overall improvement of skin parameters, we were interested in how the application of cosmetic formulations interferes with the variation of skin parameters in the morning or in the evening. During three months, the study participants applied a cosmetic formulation without (placebo) and with 3% of the active ingredient twice daily on the face. At 28, 56 and 84 days, the skin parameters were assessed in the morning and 12 h later in the evening of the same day.

**Figure 4:**
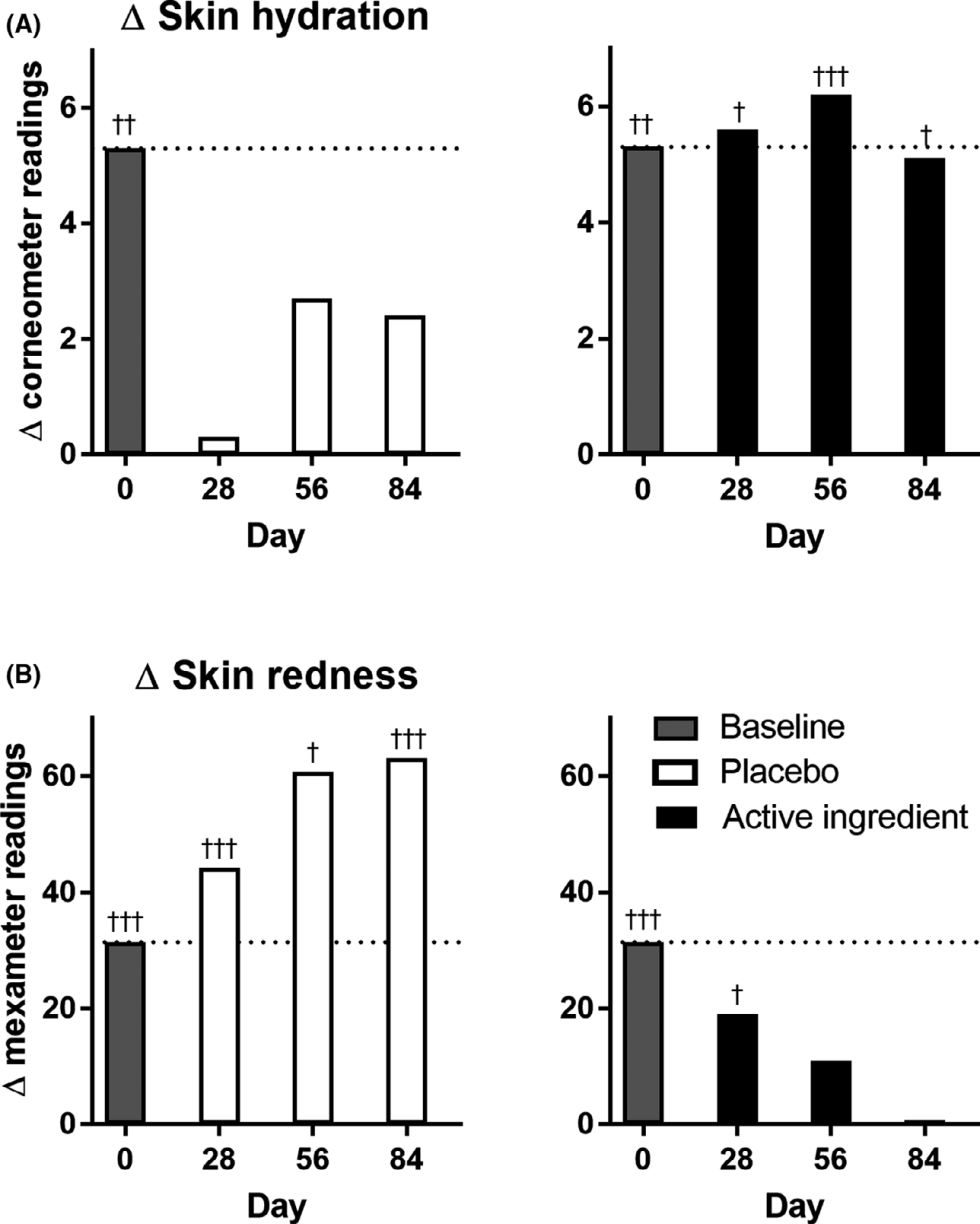
Differences of the morning and evening values at each measurement day. A: Differences in skin hydration between morning and evening values vary with placebo treatment while stay constant with active ingredient. B: Differences in skin redness increase with placebo application but being reduced with active ingredient application. †: significance over the corresponding morning value of the same day. One symbol: *P* < 0.05; two symbols: *P* < 0.01; three symbols: *P* < 0.001; unpaired Student's *t*‐test.

Skin hydration (Fig. 3A) overall increased by up to 30% in the morning and 28% in the evening at day 84 for placebo and 40% in the morning and 27 % in the evening for the active ingredient. Significance between both treatment groups was reached at day 56 for the evening values (*P* < 0.05; at day 28 *P* = 0.08). For all measurement days, skin hydration was significantly higher in the evening than in the morning for the active ingredient group, which was not the case for the placebo‐treated group. Although the maximum difference between morning and evening value for the active ingredient group resembles that of the baseline (Fig. 4A), the placebo group did not show any difference at day 28 and a reduced difference at days 56 and 84. As a result, the naturally occurring circadian change in skin hydration is preserved with the formulation containing the active ingredient whereas disturbed by just using the placebo formulation.

Skin redness increased by up to 14% in the placebo group (Fig. 3B). In contrast, the evening value for skin redness continuously decreased to the level of the morning value in case of the active ingredient (Fig. 4B).

Because of the content of the strong anti‐oxidants ethyl ferulate and carnosic acid, the active might prevent light‐induced ROS formation and DNA/cell damage with subsequent inflammatory reactions leading to an increased microcirculation. This results in a reduction of light‐induced skin redness.

The experiments show that the incorporation of an active ingredient into a basic cosmetic formulation can preserve the circadian rhythm of skin hydration and reduce the daytime skin reddening reaction. The active ingredient can stimulate the positive circadian clock genes after UVB impact and support DNA protection.

### Final discussion: Can we draw circadian conclusions from the data?

Circadian skin changes were quantified in several publications. It turns out that the circadian changes are dependent on the body site they are investigated and large time shifts may occur comparing the face skin with that of the forearm. Controversy results with significant or non‐significant changes make the interpretation of the results very difficult. The ability to have a circadian readout on skin parameters may also vary from one subject panel to the other, where we have not even mentioned the different places of life and ethnicities, habits and hygiene routines. As such, also the interpretation of our study results can only provide a limited view of reality. In our study, we found that the active ingredient consisting of the UV‐protecting ethyl ferulate and the strong antioxidant carnosic acid protected and supported the circadian rhythm of the skin. Ethyl ferulate, a modified version of ferulic acid from rice, has a broadband absorption spectrum in the UV‐B and UV‐A area [32]. This enables the molecule either to absorb UV‐light or to rapidly eliminate UV‐induced ROS on the site of their generation. Ethyl ferulate acts rather like a cellular sunscreen than a conventional SPF as it will not increase the SPF of a cosmetic sunscreen formulation. The advantage is that it can eliminate residual radiation where it would be most harmful. To eliminate excess and secondary produced ROS, for example from the metabolism, inflammatory reactions or high energy visible light, rosemary extract, especially carnosic acid and carnosine efficiently eliminate reactive oxygen species [33]. Interestingly, the combination of the ingredients of the active ingredient is able to stabilize the expression of 8‐oxo guanidine glycosylase after UV‐B impact in keratinocytes, facilitating repair of oxidatively damaged DNA and recover faster from circadian phase shift. An additional study revealed a significantly faster recovery from UV‐induced erythema when using a formulation containing the active compared to placebo or non‐treated skin. The potent *in vivo* ROS scavenging activity after UV irradiation of skin has also been reported elsewhere [34]). Although conventional cosmetic formulations may interfere in the circadian cycle of skin hydration, the active ingredient preserves this natural oscillation in skin hydration and as such supports the proper skin function.

## Acknowledgement

The presented studies have been paid by and performed for RAHN AG, Switzerland.
